# Genome-Wide Isoform Switching Reveals SR45-Mediated Splicing Control of *Arabidopsis* Leaf Senescence

**DOI:** 10.3390/ijms26199784

**Published:** 2025-10-08

**Authors:** Mohammed Albaqami, Ghaydaa Osamah Almaghrabi

**Affiliations:** 1 Department of Botany and Microbiology, College of Science, King Saud University, Riyadh P.O. Box 2455, Saudi Arabia; 2 Department of Biological Sciences, Faculty of Science, King Abdulaziz University, Jeddah P.O. Box 80200, Saudi Arabia

**Keywords:** leaf senescence, isoform switching, alternative splicing, splicing factors, SR45, *Arabidopsis thaliana*

## Abstract

Leaf senescence is the final, programmed stage of leaf development, marked by nutrient remobilization and tightly regulated molecular events. Although alternative splicing has emerged as a major regulator of plant development, its role in isoform switching during leaf aging remains poorly understood. To address this, we conducted a genome-wide analysis of isoform switching in *Arabidopsis*, leveraging publicly available RNA-seq data from mature (16-day-old) and senescent (30-day-old) leaves, analyzed with the *IsoformSwitchAnalyzeR* package. Between these two developmental stages, we identified 269 genes exhibiting 377 significant isoform switches collectively predicted to alter protein localization, coding potential, and transcript stability. Experimental validation confirmed predicted switching at the *PUS3* (*Pseudouridine Synthase 3*) locus, with sequence analysis revealing an age-dependent shift from mitochondrial-targeted to cytoplasmic isoforms. Gene Ontology enrichment analysis of switching genes revealed 82 significant terms, prominently associated with metabolism, gene expression, developmental regulation, and stress responses. Interestingly, we found nearly one-third of switching genes to overlap with known targets of the splicing factor SR45, with enrichment in pathways related to nucleotide and amino acid metabolism, energy production, and developmental processes. Correspondingly, dark-induced senescence assays revealed accelerated senescence in the *sr45* mutant, confirming SR45′s role in regulating leaf aging. Specific complementation of SR45′s two isoforms revealed contrasting functions, with SR45.1 restoring normal senescence timing while SR45.2 failed to complement. Taken together, our findings demonstrate that differential isoform usage, orchestrated by specific splicing regulators, plays a critical role in leaf aging. This insight opens new avenues for manipulating senescence and engineering stay-green traits in crops.

## 1. Introduction

Leaf senescence is the final, genetically programmed stage of leaf development and is crucial for plant fitness, nutrient recycling, and reproductive success. It is characterized by a highly coordinated transition from anabolic to catabolic processes, initiated with the dismantling of chloroplasts to remobilize nitrogen and carbon to sink tissues [1,2,3]. Morphologically, this process is visibly marked by chlorophyll degradation and leaf yellowing. While driven primarily by age, senescence is also accelerated by a variety of environmental stressors [4,5,6]. Elucidation of the regulatory networks controlling senescence is critically important for agriculture, as the timing of senescence is a major determinant of crop yield, grain quality, and overall productivity.

The molecular initiation of senescence is driven by extensive transcriptional reprogramming, with 10–16% of *Arabidopsis* genes exhibiting differential expression during aging [7]. This global shift is coordinated by senescence-associated genes (SAGs), regulated through the activation or repression of key transcription factors (TFs). The NAC and WRKY TF families serve as central regulators, integrating signals from pathways such as ethylene signaling to modulate SAG expression [8,9,10,11]. These regulators include both senescence-promoting TFs (e.g., ORE1, AtNAP, WRKY53) that accelerate aging and negative regulators (e.g., JUB1 and certain NAC troika members) that delay the process [12,13,14,15,16]. Further complexity arises from hormonal integration, wherein bHLH TFs (MYC2/3/4) link jasmonate signaling to senescence-associated transcriptional networks [17,18].

While transcriptional control is central to leaf senescence, post-transcriptional regulation, particularly alternative splicing (AS), provides a critical additional layer of complexity by generating functionally distinct isoforms from single genes. Numerous senescence regulators undergo AS, often producing isoforms with antagonistic functions. For instance, an intron-retaining variant of *PtRD26* acts as a dominant-negative repressor of NAC TFs, thereby delaying senescence [19]. Similarly, alternative polyadenylation of *ERF4* generates activator and repressor isoforms that oppositely regulate reactive oxygen species homeostasis and senescence progression [20,21]. This regulatory principle is conserved across species; in rice, both the canonical *ONAC054* and its alternatively spliced variant promote senescence [22]. In addition, the importance of the splicing machinery itself is highlighted by the report that the *Arabidopsis* spliceosomal component U2B″ delays senescence by modulating jasmonate signaling through preferential selection of the *JAZ9β* isoform [23]. These examples center AS as a key mechanism by which senescence timing is fine-tuned; nonetheless, the establishment of a comprehensive, genome-wide view of isoform switching during natural aging remains an open area of investigation.

Isoform switching refers to a change in the relative abundance of transcript isoforms from a given gene, often driven by AS events such as intron retention, exon skipping, and alternative donor or acceptor site usage [24]. Such switches can alter coding potential, resulting in proteins with modified domains, subcellular localization, or stability, or generate noncoding transcripts subject to degradation [25,26]. Beyond protein-level effects, isoform switches can also modulate mRNA translation efficiency, regulatory element composition, and interactions with other RNAs or proteins [27,28,29]. In this way, isoform switching enables the selective, spatiotemporally defined deployment of functionally distinct isoforms to regulate complex traits. In plants, isoform switching is increasingly recognized as a dynamic regulatory mechanism shaping developmental transitions and stress responses [30,31,32]. In the context of aging, isoform switching offers a versatile means of reprogramming cellular functions, making it a potentially critical yet underexplored layer in the control of leaf senescence.

Splicing regulators, particularly serine/arginine-rich (SR) proteins, are central modulators of AS that shape transcript diversity and gene expression outcomes [33,34]. Canonical SR proteins contain RNA recognition motifs (RRMs) and a C-terminal RS domain, which facilitate pre-mRNA binding and spliceosome recruitment [35]. *Arabidopsis* SR45 is an atypical SR protein with a unique domain arrangement, an RRM flanked by RS domains on both sides, that enables dual functionality in splicing regulation and mRNA surveillance through interactions with exon junction complex components [36,37]. Through these activities, SR45 regulates diverse processes including development (e.g., root growth, flowering time, and leaf morphology) and responses to abiotic stresses such as salinity and light [37,38,39,40,41]. Notably, previous transcriptome-wide identification of SR45-associated RNAs revealed over 4,000 target transcripts, including numerous abscisic acid (ABA) signaling genes and, unexpectedly, many intronless transcripts, a finding that suggests SR45 to have a broader role in mRNA processing beyond conventional splicing [42]. The functional diversification of SR45 is further exemplified by its two alternatively spliced isoforms, SR45.1 and SR45.2. Despite differing by only seven amino acids, these isoforms exhibit distinct, often antagonistic roles in development and stress responses [40,41,43,44]. Overall, the roles of SR45 in development and stress are well-established, but its potential function in regulating isoform switching during leaf aging has not been explored.

In parallel, there is growing evidence for the role of AS in plant development, but its contribution to isoform switching during leaf senescence remains unclear. Here, we performed a genome-wide analysis in *Arabidopsis* exploring AS associated with leaf senescence, which identified 269 genes with 377 significant isoform switches between mature and senescent leaves. As a representative case, we validated the switching identified at the *PUS3* locus; notably, the isoform that increases with age is predicted to localize to the cytoplasm, while the declining isoform is mitochondrial-targeted. More broadly, ~30% of switching genes overlapped with known targets of the splicing regulator SR45. Subsequent functional assays not only confirmed a role for SR45 in senescence but also uncovered its isoforms to have specific, contrasting functions: SR45.1 delayed senescence, while SR45.2 failed to complement the mutant phenotype. Together, our findings establish SR45-dependent isoform switching as a key post-transcriptional mechanism that fine-tunes the progression of leaf senescence, opening new avenues for manipulating aging in plants.

## 2. Results

### 2.1. Isoform Switching During Arabidopsis Leaf Senescence

**Figure 1:**
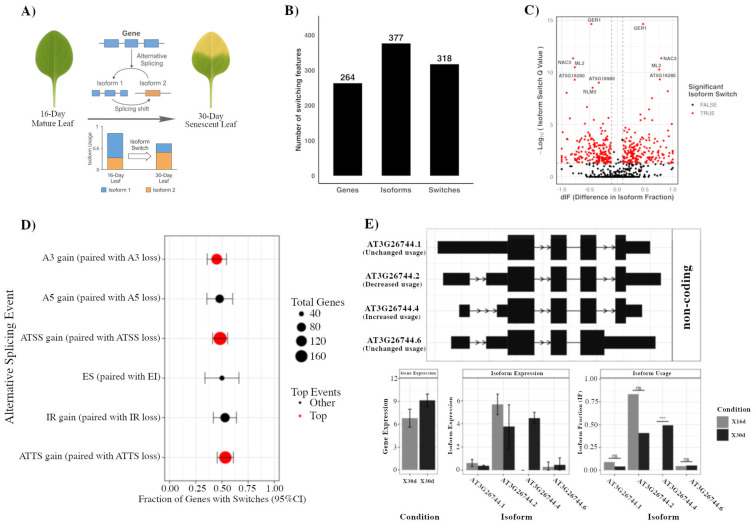
Isoform switching during *Arabidopsis* leaf senescence. (**A**) Schematic of the experimental comparison of 16-day-old mature leaves to 30-day-old senescent leaves. (**B**) Number of expressed genes, detected isoforms, and significant isoform switches. (**C**) Volcano plot of difference in isoform fraction (dIF) versus adjusted *p*-value, with significant switches (|dIF| ≥ threshold, FDR ≤ 0.05) highlighted in red. (**D**) Distribution of AS event types among switched genes, showing the fraction of genes (±95% CI) with top-ranked events in red. (**E**) Example of a switch in *ICE1*, including transcript models, gene and isoform expression, and isoform usage changes between 16-day and 30-day leaves. (*** *p* < 0.001, ns, not significant).

To determine whether isoform-level regulation contributes to leaf aging, we analyzed RNA-seq datasets comparing mature (16-day) and senescent (30-day) *Arabidopsis* leaves (Figure 1A). Isoform usage was quantified with the *IsoformSwitchAnalyzeR* framework, in which the relative contribution of each isoform to total gene expression is expressed as an Isoform Fraction (*IF*). Differences in *IF* between developmental stages (*dIF* = *IF*_30d_ *– IF*_16d_) were used to identify significant switching events. This analysis uncovered 264 genes that exhibited isoform switching in senescent versus mature leaves, comprising 377 isoforms and 318 significant events that passed stringent thresholds (|dIF| ≥ 0.1; FDR < 0.05) (Figure 1B,C and Table S2). As an illustrative example, the *ICE1* locus displayed a pronounced switch: *Isoform 4* was strongly upregulated in senescent leaves even as the total gene expression remained unchanged (Figure 1E). This case highlights a central hypothesis of our dataset, that isoform reprogramming can fine-tune gene function independently of overall transcript abundance.

Examination of the distribution of splicing event classes identified the most frequent types to be alternative 3′ splice sites (A3), alternative transcription start sites (ATSS), and alternative transcription termination sites (ATTS) (Figure 1D). These events all typically occurred as paired gain–loss transitions between isoforms. Statistical enrichment testing confirmed that no individual event type was significantly over-represented (Figure S1A), indicating that the dominance of A3, ATSS, and ATTS classes reflects widespread but not preferential usage. Further assessment of the predicted functional consequences of isoform switching (Figure S1B) determined no outcome category to reach significance (FDR > 0.05), but found isoform switches to frequently involve intron retention, nonsense-mediated decay sensitivity, and open reading frame length variation, pointing to potential effects on transcript stability and coding capacity at the individual-gene level. Collectively, these results demonstrate that isoform switching is a pervasive regulatory layer during *Arabidopsis* leaf senescence, dominated by A3, ATSS, and ATTS transitions, and capable of reshaping transcript coding potential or stability in a gene-specific manner without necessarily altering overall gene expression.

### 2.2. Isoform Switching Uncoupled from Gene Expression Alters Protein Localization

**Figure 2:**
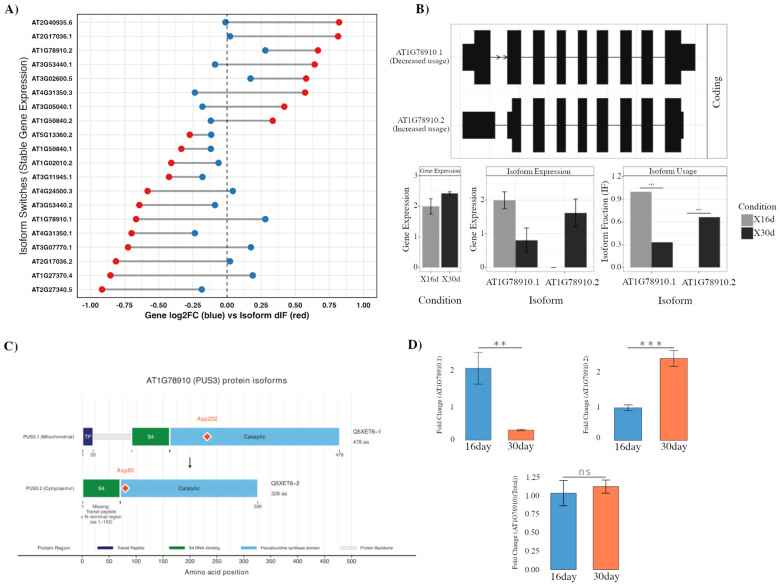
Top isoform switches lacking total gene expression change and characterization of the isoform switch in *AT1G78910* (*PUS3*). (**A**) Top 20 genes showing significant isoform switching (red, dIF) without corresponding change in total gene expression (blue, log_2_ fold-change) between 16-day and 30-day leaves. (**B**) The *PUS3* isoform switch, showing transcript models, gene and isoform expression, and isoform usage changes. (**C**) Protein domain architecture of the two *PUS3* isoforms, highlighting the presence or absence of the mitochondrial targeting sequence, differences in domain organization, and catalytic residue positions. (**D**) qPCR validation of *PUS3* expression, showing total gene and isoform-specific expression changes between 16-day and 30-day leaves, confirming the RNA-seq–detected switch. Asterisks indicate statistically significant differences determined by Student’s *t*-test (** *p* < 0.01, *** *p* < 0.001, n.s., not significant).

While many isoform switches were accompanied by overall shifts in gene expression, we identified a subset of genes in which isoform usage changed markedly despite the corresponding total transcript abundance remaining stable. This phenomenon underscores the potential for isoform-level reprogramming to fine-tune gene function independently of differential gene expression. To explore this regulatory principle, we ranked all significant isoform switches by the magnitude of differential isoform fraction relative to their gene-level log_2_ fold-change; the top 20 cases are presented as illustrative examples (Figure 2A).

One of the most striking isoform shifts was observed for *AT1G78910* (*PUS3*), which encodes a pseudouridine synthase. Transcript-level analysis showed a clear redistribution from the *PUS3.1* isoform toward the *PUS3.2* isoform during senescence, even as overall gene expression remained largely unchanged (Figure 2A,B). Protein domain modeling indicated the two isoforms to differ substantially: PUS3.1 encodes a longer protein containing a predicted N-terminal mitochondrial targeting sequence, whereas PUS3.2 lacks this sequence and instead produces a cytoplasmic isoform. Both isoforms retain the S4 RNA-binding domain and catalytic core, including the conserved aspartate residue essential for enzymatic activity, but their subcellular targeting and regulatory potential are predicted to diverge (Figure 2C).

To validate this switching, we performed isoform-specific qPCR analysis. Consistent with the RNA-seq results, total *PUS3* transcript level showed no significant change between mature and senescent leaves, whereas *PUS3.2* was significantly upregulated and *PUS3.1* concomitantly reduced at 30 days (Figure 2D). These findings confirm the presence of a functionally relevant isoform switch at the *PUS3* locus during senescence and highlight how isoform switching can reprogram protein localization and function independent of a change in total gene expression.

### 2.3. Functional Enrichment of Isoform Switching Genes

**Figure 3:**
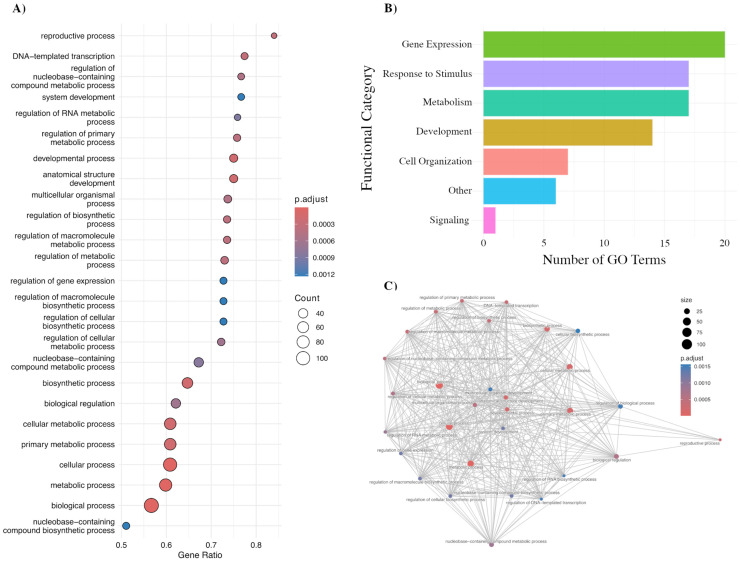
Gene Ontology (GO) enrichment analysis of switching genes during *Arabidopsis* leaf senescence. (**A**) Top 25 enriched Biological Process GO terms identified by GSEA, ranked by gene ratio. Dot size represents the number of genes associated with each term, and color indicates adjusted *p*-value. (**B**) Functional categorization of enriched GO terms, showing the distribution across major biological categories. (**C**) GO term network visualization, where nodes represent enriched GO terms and edges indicate genes shared between terms. Node size reflects gene count and node color represents adjusted *p*-value.

To explore the biological significance of isoform switching during leaf senescence, we conducted GO enrichment analysis on the impacted genes. This identified 82 significantly enriched GO terms (Table S3) spanning broad aspects of plant biology, of which the top 25 Biological Process terms are presented in Figure 3A, including highly significant categories such as *cellular process* (GO:0009987; adjusted *p* = 3.6 × 10^−7^), *metabolic process* (GO:0008152; adjusted *p* = 9.9 × 10^−6^), *biological process* (GO:0008150; adjusted *p* = 1.3 × 10^−5^), and *cellular metabolic process* (GO:0044237; adjusted *p* = 2.0 × 10^−4^). These broad categories highlight that isoform switching affects genes central to metabolic activity, cellular organization, and developmental regulation. When grouped into higher-level functional categories (Figure 3B and Table S4), enriched terms were dominated by gene expression (21 terms), response to stimulus (18 terms), metabolism (18 terms), and development (17 terms), underscoring the role of isoform reprogramming in controlling transcriptional networks, metabolic adjustments, and developmental progression during senescence.

To better visualize functional themes, we constructed a GO term similarity network based on the enriched Biological Process terms (Figure 3C and Table S5), which revealed three prominent hubs of interconnected categories. The first centered on core metabolic and biosynthetic processes, including metabolic process, biosynthetic process, and regulation of biosynthetic process. A second hub was dominated by terms related to gene expression regulation, such as regulation of RNA biosynthetic process, transcription, DNA-templated processes, and regulation of gene expression. The third hub grouped developmental processes, including system development, multicellular organism development, and developmental process. These hubs, defined by high connectivity and significance, highlight that isoform switching during leaf senescence preferentially affects central regulatory nodes in metabolism, transcriptional control, and development.

### 2.4. SR45 Targets Overlap with Isoform-Switching Genes

**Figure 4:**
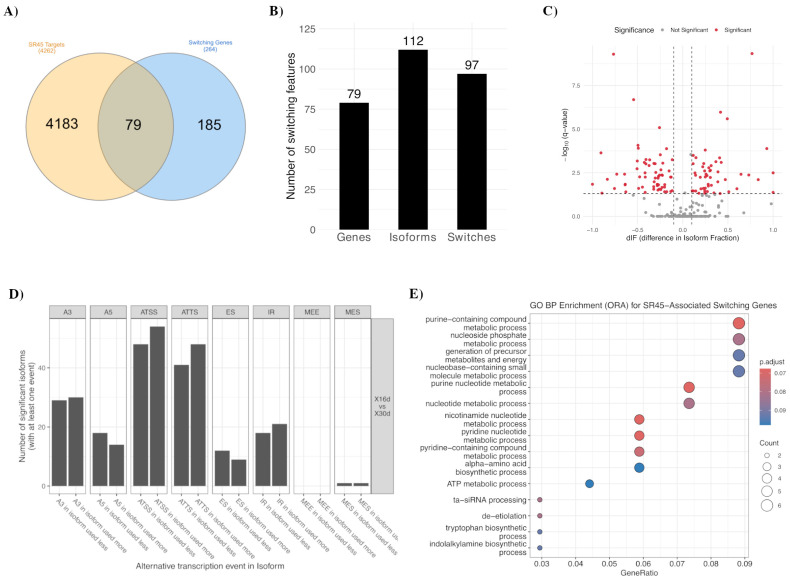
Overlap between SR45 targets and genes that undergo isoform switching during *Arabidopsis* leaf senescence. (**A**) Venn diagram showing the intersection between previously identified SR45 target genes and senescence-associated switching genes, revealing 79 overlapping genes. (**B**) Number of genes, isoforms, and significant isoform switches within the overlapping set. (**C**) Volcano plot of isoform switching in SR45-associated genes, showing the difference in isoform fraction (dIF) against adjusted *p*-value; significant switches (FDR ≤ 0.05, |dIF| ≥ threshold) are highlighted in red. (**D**) Distribution of alternative transcription and splicing events among SR45-associated switching isoforms, categorized by event type (A3, A5, ATSS, ATTS, ES, IR, MEE, MES). (**E**) GO Biological Process enrichment for SR45-associated switching genes, with dot size indicating the number of genes per term and color the adjusted *p*-value.

Splicing factors, particularly SR proteins, are key regulators of transcriptome diversity in *Arabidopsis*. Given SR45′s important roles in development and stress response, we hypothesized that it might directly regulate splicing events during senescence. To test this, we assessed the overlap between genes subject to senescence-associated isoform switching (n = 264) and experimentally defined SR45-bound transcripts (n = 4262) from a published RIP-seq dataset [42]. This revealed a significant overlap of 79 genes representing a focused subset of switching candidates likely under direct SR45-mediated post-transcriptional regulation (Figure 4A and Table S6), which together accounted for 112 specific isoforms and 97 individual switching events (Figure 4B). Visualization by volcano plot highlighted the pronounced isoform fraction changes (dIF) within this overlapping gene set (Figure 4C). Classification of the underlying splicing event types revealed ATSS, ATTS, and A3 to all predominate, a pattern consistent with the global trends observed in the full senescence dataset (Figure 4D).

To assess the specificity of this overlap, we compared our data to targets of AtGRP7 (n = 2453), a distinct RNA-binding protein involved in post-transcriptional regulation [45]. This control analysis revealed a minimal overlap, with only ten switching genes found among AtGRP7 targets, versus the 79 for SR45 (Figure S3 and Table S7). This near-order-of-magnitude difference demonstrates that senescence-associated isoform switching is non-random and is particularly enriched for genes within the SR45 splicing network.

Subsequent functional enrichment analysis of the SR45-associated switching genes revealed significant overrepresentation in metabolic and nucleotide-related processes (Figure 4E and Table S8), most notably ATP metabolism, RNA processing, amino acid biosynthesis, and light signaling, as well as indole/tryptophan biosynthetic pathways. These functional categories align with well-established hallmarks of senescence, which is characterized by extensive metabolic reprogramming wherein energy turnover, nucleic acid metabolism, and secondary metabolite biosynthesis are central. Together, these findings define a focused subset of transcripts whose alternative splicing during senescence is likely directly modulated by SR45, and thereby highlight the role of SR45 in fine-tuning pathways critical for energy balance and resource allocation during leaf aging.

### 2.5. SR45 Regulates DILS in an Isoform-Specific Manner

**Figure 5:**
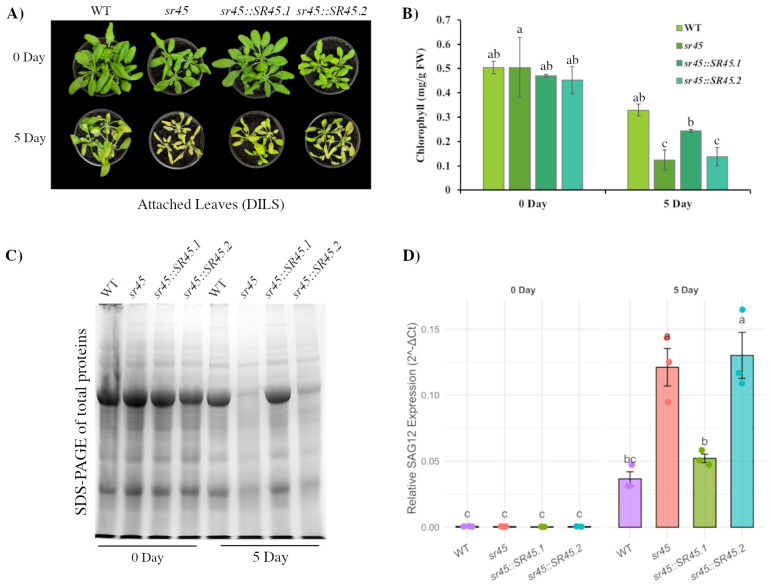
Phenotypic and molecular characterization of SR45 lines during dark-induced leaf senescence (DILS). (**A**) Representative images of attached leaves from wild-type (WT), *sr45*, *sr45::SR45.1*, and *sr45::SR45.2* plants at 0 and 5 days of dark incubation. (**B**) Chlorophyll content per gram fresh weight (FW) at 0 and 5 days, showing significant reductions in *sr45* and *sr45::SR45.2* compared to WT and *sr45::SR45.1*. Different letters indicate statistically significant differences (*p* < 0.05, one-way ANOVA, Tukey’s HSD). (**C**) SDS-PAGE profiles of total proteins extracted from leaves at 0 and 5 days, showing overall protein degradation during senescence. (**D**) Relative expression of the senescence-associated gene *SAG12* as measured by qPCR in WT and transgenic lines at 0 and 5 days, normalized to an internal control (*ACT2*). Bars represent mean ± SE of biological replicates; different letters indicate statistically significant differences (*p* < 0.05).

The significant overlap between senescence-associated isoform switches and SR45 targets suggested a role for this splicing factor in leaf aging. To test this, we assessed the functional consequence of SR45 loss of function mutation and isoform-specific overexpression on the progression of DILS (Figure 5). Moreover, given that the SR45.1 and SR45.2 isoforms exert distinct and frequently antagonistic effects on other developmental processes, we asked if these isoforms also differentially regulate senescence.

Phenotypic analysis revealed *sr45* mutant leaves to exhibit accelerated senescence, specifically enhanced yellowing under dark treatment compared to the WT (Figure 5A). Chlorophyll quantification confirmed this observation, with significantly greater loss of pigment in *sr45* relative to WT by day 5 (Figure 5B). In complementation tests, overexpression of SR45.1 partially restored chlorophyll retention, but in stark contrast, overexpression of SR45.2 failed to rescue the phenotype, instead resembling the *sr45* mutant. These findings were corroborated by protein degradation assays (SDS–PAGE), which showed *sr45* mutants to undergo accelerated global protein loss. This phenotype was again fully rescued in SR45.1 complementation lines but persisted in SR45.2 lines (Figure 5C). Finally, qPCR analysis of the senescence marker *SAG12* showed its marked induction in *sr45*, which was suppressed by SR45.1 but not by SR45.2 (Figure 5D), reinforcing the isoform-specific role of SR45.1 as a negative regulator of senescence.

To assess the specificity of SR45′s role, we additionally analyzed DILS responses in mutants of two other SR proteins, *sr30* and *sr33*. Neither mutant displayed a senescence phenotype pronouncedly different from the WT (Figure S4), indicating that among these related factors, SR45 has a unique role in modulating senescence. Together, these experiments establish SR45 as a central negative regulator of DILS, with functionally distinct contributions from its isoforms: SR45.1 provides enhanced protection against premature senescence while SR45.2 appears to lack this function. These findings highlight the capacity for splice variants to encode a critical regulatory divergence.

## 3. Discussion

Leaf senescence constitutes one of the most dramatic cellular reprogramming events in plant development, yet the post-transcriptional mechanisms governing this transition remain poorly understood. Here, we uncover isoform switching as a pervasive post-transcriptional layer shaping this process. Using *IsoformSwitchAnalyzeR*, we identified 264 genes that undergo significant isoform reprogramming between mature and senescent leaves, for which most switches occur independent of a change in total gene expression. These events were dominated by alternative transcription start sites (ATSS), transcription termination sites (ATTS), and alternative 3′ splice sites (A3), classes of AS that are frequently predicted to alter coding potential, stability, or protein localization. Functional enrichment pointed to metabolic and nucleotide-related pathways as key targets, linking splicing regulation to senescence-associated metabolic remodeling. Integration with SR45 target datasets and functional assays in both *sr45* mutants and isoform overexpression lines established SR45 as a central regulator of leaf senescence with isoform-specific contributions; that is, SR45.1 but not SR45.2 delays premature senescence. Collectively, these results highlight isoform switching as a critical mechanism for fine-tuning gene function during leaf aging.

Although AS has long been implicated in plant developmental transitions, its role in leaf senescence has been documented primarily through studies of individual genes rather than genome-wide surveys. For example, isoform switches in key senescence regulators such as PtRD26, ERF4, ONAC054, and U2B″ have been shown to modulate DNA binding, redox homeostasis, or hormone signaling, and thereby ultimately alter the pace of senescence [19,20,21,22,23]. However, the broader prevalence and functional significance of isoform-level regulation across the transcriptome in the senescence context remains unexplored. Our study addresses this gap by demonstrating that isoform switching is both pervasive and functionally consequential during *Arabidopsis* leaf senescence; moreover, such switching leads to changes in protein localization, coding potential, and transcript stability, as exemplified by the case of *PUS3*, wherein the age-associated switch between isoforms with and without a mitochondrial targeting sequence occurs independent of total transcript abundance. These findings demonstrate that the proteome of senescing cells can be fundamentally reshaped at the isoform level without need for wholesale changes in gene expression.

GO enrichment analyses of both the global isoform-switching set and the SR45-overlapping subset consistently highlighted metabolic and nucleotide-related processes, pathways long recognized as central to leaf senescence. Previous studies have shown that senescence is accompanied by a reprogramming of energy metabolism, macromolecule degradation, and nutrient remobilization [46]. Our data extend this view by demonstrating that isoform switching contributes an additional post-transcriptional layer to this remodeling, with predicted effects on protein coding potential and subcellular targeting. The *PUS3* locus provides a striking example: while the canonical isoform encodes a mitochondrial protein, the senescence-associated isoform redirects the protein to the cytoplasm. This switch implies a shift away from mitochondrial RNA modification toward cytoplasmic functions, potentially aligning with the cytoplasmic RNA processing and nutrient reallocation that characterize late stages of senescence. These findings fundamentally expand our understanding of senescence regulation by revealing that the cell employs isoform switching not merely as a passive consequence of aging, but as an active regulatory strategy to orchestrate the complex metabolic and cellular reorganization required for efficient nutrient recovery and programmed cell death.

Among splicing machinery, SR45 functions as a central hub, its effects mediated through specific interactions with core spliceosomal proteins (e.g., U1-70K, U2AF35) and regulatory complexes (e.g., SKIP, SUA, ASAP) [47,48,49]. Transcriptome-wide analyses have demonstrated SR45 to associate with thousands of RNAs, with notable enrichment for transcripts involved in ABA signaling [42], ABA being a well-characterized promoter of leaf senescence. Crucially, this phytohormone also acts upstream to stabilize SR45 protein level, and the *sr45* mutant is hypersensitive to ABA [38], establishing a direct molecular link between hormonal signaling and the splicing regulatory hub. Our discovery of an accelerated senescence phenotype in the *sr45* mutant provides direct functional validation of this model and confirms that SR45 is an essential orchestrator of the transcriptome remodeling required for controlled leaf aging.

SR45 is encoded by a single gene from which AS generates two major isoforms, SR45.1 and SR45.2, that differ by only seven amino acids. This short insertion in SR45.1 introduces serine and threonine residues that serve as potential phosphorylation sites, providing a molecular basis for isoform-specific regulation [43,44]. Previous work has established the functional divergence of these isoforms, namely that they complement different developmental and stress response defects in the *sr45* mutant [40,41,43,44]. Our study further reveals this functional divergence to be critically important in senescence regulation: SR45.1 complementation restored WT senescence timing, whereas SR45.2 failed to prevent premature aging. These findings demonstrate how minimal sequence variation at phosphorylation-sensitive sites can generate functionally distinct regulatory outputs. Given that SR45 activity and stability are themselves modulated by ABA-mediated phosphorylation, our data suggest a model whereby differential modification of these two isoforms provides a precise mechanism to couple hormonal signaling with the transcriptome remodeling required for senescence. Building on our validation of isoform switching at the *PUS3* locus, future work should extend isoform-level quantification of additional SR45 targets in SR45.1 and SR45.2 overexpression lines as well as the *sr45* mutant to further refine mechanistic insight.

## 4. Materials and Methods

### 4.1. Plant Materials and Growth Conditions

All *Arabidopsis thaliana* lines used in this study were in the Columbia (Col-0) ecotype, with the wild-type (WT) Col-0 serving as the genetic background control. The *sr45* loss-of-function mutant (Salk_004132) was previously isolated and described by Ali et al. [37]. Isoform-specific complementation lines (*sr45::SR45.1* and *sr45::SR45.2*) were generated using the *CaMV 35S* promoter in the *sr45* mutant background and characterized as previously described [43]. T-DNA insertion mutants for the splicing factors SR33 (*SALK_035975*) and SR30 (*SALK_058566*) were obtained from the Arabidopsis Biological Resource Center (ABRC), and were genotyped to confirm homozygous T-DNA insertions. For experimental uniformity, all seeds originated from the same planting batch. Seeds were surface-sterilized with 15% (v/v) sodium hypochlorite and stratified at 4 °C for 72 h, then germinated on plates containing 0.5× Murashige and Skoog basal salt medium with 0.8% (w/v) agar and grown for seven days in a controlled growth chamber (22 °C, 120 μmol m^−2^ s^−1^ light intensity, 12 h light/12 h dark cycle). Seedlings were finally transplanted into a peat-based potting soil mixture and maintained under the same environmental conditions for subsequent experiments. Plants were irrigated with distilled water every 2–3 days to maintain consistent soil moisture without waterlogging.

### 4.2. RNA-Seq Data Acquisition and Processing

Publicly available RNA-seq data from *Arabidopsis thaliana* (Col-0) leaves were obtained from Woo et al. [50] (GEO: GSE43616; SRA: SRP018034). From the full 14-time-point series, we selected day 16 (mature) and day 30 (senescent) leaves, each with two biological replicates (SRR2079777, SRR2079791, SRR2079784, SRR2079798). In the original study, total RNA was extracted from *Arabidopsis* leaves using WelPrep (WelGENE, Gyeongsan-si, Korea). Sequencing on the Illumina HiSeq 2000 platform generated ~20–29 million paired-end reads (101 bp) per sample. Raw reads were quality-checked with FastQC v0.11.9 and summarized using MultiQC v1.29, which confirmed high per-base quality (mean Phred > Q30) and minimal adapter contamination. The *Arabidopsis* Araport11 transcriptome (TAIR10) from Ensembl Plants was indexed with Salmon v1.10.3, and transcript abundance was quantified using bias correction options (--gcBias, --seqBias, --posBias) and --validateMappings. Mapping rates ranged from 83.6% to 88.5%, and quantification outputs (quant.sf files) were used for subsequent isoform switching analyses.

### 4.3. Isoform Switching Analysis

Transcript quantification files from Salmon were imported into R (v4.4.2) for isoform switching analysis using the *IsoformSwitchAnalyzeR* package (v2.3.0) [51]. Transcript structures and gene models were defined based on the Araport11 annotation (GTF) [52]. After importing quantification data with importIsoformExpression and importRdata, transcripts with low expression (average TPM < 1) or short coding sequences (< 300 nt) were removed. Isoform switches between day 16 (mature) and day 30 (senescent) samples were detected with isoformSwitchTestDEXSeq, applying a differential isoform fraction cutoff of ≥ 0.1 and a false discovery rate (FDR) < 0.05. Functional consequences of isoform switches were predicted using integrated tools for coding potential (CPAT v1.2.4), open reading frame analysis, protein domain annotation (Pfam v35.0), and signal peptide prediction (SignalP v5.0). Results were summarized using extractSwitchSummary and extractConsequenceSummary to identify genes with significant isoform switching and associated structural or functional changes.

### 4.4. Gene Ontology (GO) Enrichment Analysis

To explore functional themes, GO enrichment analysis was conducted in R (v4.4.2) using the *clusterProfiler* package (v4.10.0). As expression-based rankings were not the focus of this study, all genes in each set (all isoform-switching genes and the subset overlapping with SR45-dependent targets) were assigned equal weights for an exploratory GSEA-style analysis to identify functional categories relevant to leaf aging. The gseGO function was applied with the org.At.tair.db annotation database, a focus on the Biological Process ontology, a minimum gene set size of 5, and an adjusted *p*-value (Benjamini–Hochberg FDR) cutoff of 0.1. Redundant terms were removed using simplify with a similarity cutoff of 0.7, and the results were visualized as dot plots of top enriched terms, functional categorization across major biological themes, and a GO term network highlighting interrelationships among enriched processes.

### 4.5. SR45 Target Overlap Analysis

*Arabidopsis* SR45 target genes were obtained from the dataset reported by [42], who identified transcriptome-wide RNA targets of SR45 using RNA immunoprecipitation followed by high-throughput sequencing (RIP-seq). To assess whether isoform-switching events in our dataset are associated with known SR45 regulation, we performed an overlap analysis between our isoform-switching genes and the reported SR45 targets. Overlaps were assessed at the gene level to capture broader biological roles and avoid underestimating overlaps that may arise at the isoform level.

### 4.6. Dark-Induced Leaf Senescence (DILS) and Chlorophyll Content Analysis

DILS was performed on 21-day-old plants grown as specified above by covering entire plants with aluminum foil; control plants were maintained under normal light in the same growth chamber. Leaves of the same developmental stage (positions five and six) were harvested at day 0 and after five days of treatment. Chlorophyll was extracted in 80% (v/v) acetone overnight at 4 °C [53] and the total chlorophyll content (mg g^−1^ fresh weight) was calculated as: (20.2 × A_646_) + (8.02 × A_663_) × extract volume (mL)/(1000 × fresh weight [g] × path length [cm]), where A_646_ and A_663_ are absorbance readings at 646 nm and 663 nm, respectively. Each biological replicate consisted of leaves collected from different plants grown under the same conditions, ensuring independence of replicates.

### 4.7. Total RNA Extraction and RT-PCR

Total RNA was extracted from 16-day-old and 30-day-old leaves, or from light- and dark-treated plants, using the RNeasy Plant Mini Kit (Qiagen, Hilden, Germany). Genomic DNA was removed with DNase I (Fermentas, Hanover, MD, USA), and first-strand cDNA was synthesized with SuperScript III reverse transcriptase (Invitrogen, Carlsbad, CA, USA) in 20 µL reactions. qPCR was performed on a LightCycler 480 (Roche, Penzberg, Germany) with SYBR Green I Master mix under the following program: 95 °C for 3 min, then 44 cycles of 95 °C for 10 s, 60 °C for 30 s, and 72 °C for 30 s. *ACTIN-2* served as the internal reference. Each genotype/treatment was analyzed in three biological replicates, each with three technical replicates. Each biological replicate consisted of leaves collected from different plants grown under the same conditions, ensuring independence of replicates. Primers (Supplementary Table S1) were designed using PRIMER3Plus (https://www.primer3plus.com).

### 4.8. Protein Extraction and SDS-PAGE

Total proteins were extracted from 21-day-old treated or control plants as described by Wessel and Flügge [54] with minor modifications. Briefly, 100 mg of frozen tissue was ground in liquid nitrogen, homogenized in 200 µL protein extraction buffer, and centrifuged at 15,000 *g* for 15 min at 4 °C. Supernatants were mixed with 1× SDS sample buffer, heated at 95 °C for 5 min, and resolved by 10% SDS-PAGE [55]. Gels were stained with Coomassie Brilliant Blue and destained before imaging.

### 4.9. Statistical Analysis

Data were analyzed in R (v4.4.2), and the results are presented as mean ± standard error (SE) from at least three biological replicates. Statistical significance was assessed by one-way or two-way ANOVA followed by Tukey’s multiple comparisons test, or by Student’s *t*-test when comparing two groups. Differences were considered significant at *p* < 0.05.

## 5. Conclusions

Our study establishes isoform switching as a pervasive and functionally critical layer of gene regulation during *Arabidopsis* leaf senescence. By integrating transcriptome-wide analyses with functional validation, we demonstrate that splicing reprogramming systematically targets metabolic pathways and redirects protein localization, exemplified by the mitochondrial-to-cytoplasmic shift of PUS3. We further identify SR45 as a master regulator orchestrating senescence-associated isoform switches through isoform-specific activities that precisely control aging timing. Collectively, our findings reveal AS as an essential regulatory mechanism coordinating the molecular choreography of leaf senescence and open new directions for understanding post-transcriptional control in plant development.

## Data Availability

Data are contained within the article or Supplementary Material.

## Supplementary Materials

The following supporting information can be downloaded at: https://www.mdpi.com/article/10.3390/ijms26199784/s1.
