# Supplementary material for: Genome-Wide Isoform Switching Reveals SR45-Mediated Splicing Control of Arabidopsis Leaf Senescence
Source: Int J Mol Sci. 2025 Oct 8;26(19):9784. doi: 10.3390/ijms26199784 (PMC12525425; doi:10.3390/ijms26199784)
Supplement: Supplementary file 1 [file ijms-26-09784-s001.zip › Supplementary_Figures.pdf]

A)

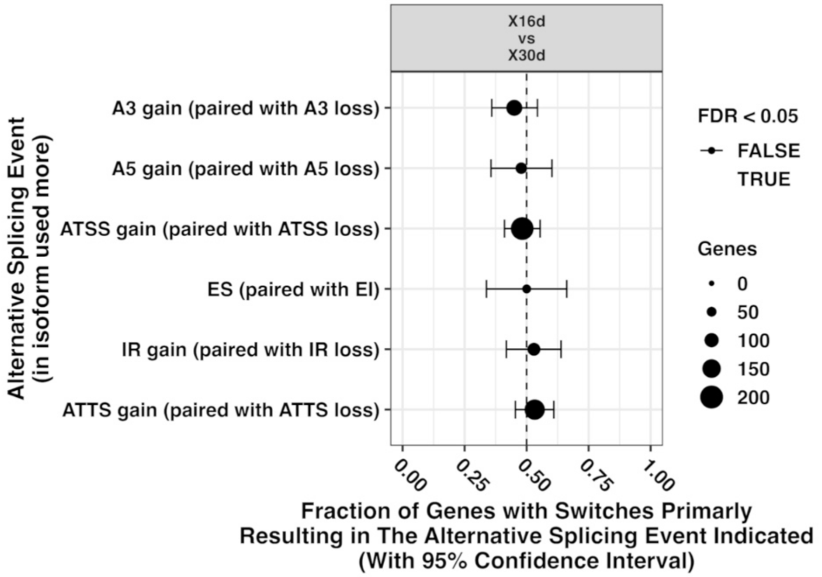

B)

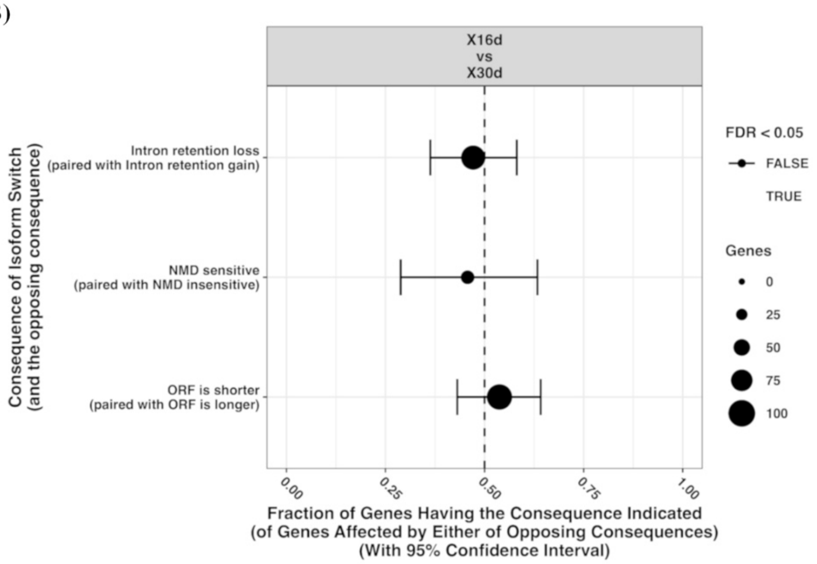

Figure S1. Alternative splicing events and predicted functional consequences of isoform switching during *Arabidopsis* leaf senescence. (A) Distribution of AS event types among genes subject to switching between 16-day and 30-day leaves. Dots represent the fraction of genes undergoing each event (±95% CI). (B) Predicted functional consequences of isoform switches, including intron retention, nonsense-mediated decay sensitivity, and changes in open reading frame length, shown as the fraction of affected genes (±95% CI). Dot color indicates statistical significance (FDR < 0.05: TRUE vs. FALSE).

A)

```
CLUSTAL O(1.2.4) multiple sequence alignment


|Q5XET6-1|PUS3_ARATH      MWKAKTCFRQIYLTVLIRRYSRVAPPPSSVIRVTNNVAHLGPPKQGPLPRQLISLPPFPG 60
|Q5XET6-2|PUS3_ARATH      ------------------------------------------------------------ 0


|Q5XET6-1|PUS3_ARATH      HPLPGKNAGADGDDGDSGGHVTAISWVKYYFEEIYDKAIQTHFTKGLVQMEFRGRRDASR 120
|Q5XET6-2|PUS3_ARATH      ------------------------------------------------------------ 0


|Q5XET6-1|PUS3_ARATH      EKEDGAIPMRKIKHNEVMQIGDKIWLPVSIAEMRISKRYDTIPSGTLYPNADEIAYLQRL 180
|Q5XET6-2|PUS3_ARATH      --------------------------------MRISKRYDTIPSGTLYPNADEIAYLQRL 28
                                                          ****************************

|Q5XET6-1|PUS3_ARATH      VRFKDSAIIVLNKPPKLPVKGNVPIHNSMDALAAAALSFGNDEGPRLVHRLDRETSGLLV 240
|Q5XET6-2|PUS3_ARATH      VRFKDSAIIVLNKPPKLPVKGNVPIHNSMDALAAAALSFGNDEGPRLVHRLDRETSGLLV 88
                          ************************************************************

|Q5XET6-1|PUS3_ARATH      MGRTKESIDYLHSVFSDYKGRNSSCKAWNKACEAMYQQYWALVIGSPKEKEGLISAPLSK 300
|Q5XET6-2|PUS3_ARATH      MGRTKESIDYLHSVFSDYKGRNSSCKAWNKACEAMYQQYWALVIGSPKEKEGLISAPLSK 148
                          ************************************************************

|Q5XET6-1|PUS3_ARATH      VLLDDGKTDRVVLAQGSGFEASQDAITEYKVLGPKINGCSWVELRPITSRKHQLRVHCAE 360
|Q5XET6-2|PUS3_ARATH      VLLDDGKTDRVVLAQGSGFEASQDAITEYKVLGPKINGCSWVELRPITSRKHQLRVHCAE 208
                          ************************************************************

|Q5XET6-1|PUS3_ARATH      ALGTPIVGDYKYGWFVHKRWKQMPQVDIEPTTGKPYKLRRPEGLDVQKGSVLSKVPLLHL 420
|Q5XET6-2|PUS3_ARATH      ALGTPIVGDYKYGWFVHKRWKQMPQVDIEPTTGKPYKLRRPEGLDVQKGSVLSKVPLLHL 268
                          ************************************************************

|Q5XET6-1|PUS3_ARATH      HCREMVLPNIAKFLHVMNQQETEPLHTGIIDKPDLLRFVASMPSHMKISWNLMSSYLV   478
|Q5XET6-2|PUS3_ARATH      HCREMVLPNIAKFLHVMNQQETEPLHTGIIDKPDLLRFVASMPSHMKISWNLMSSYLV   326
                          **********************************************************
```

B)

```
CLUSTAL O(1.2.4) multiple sequence alignment


1       TTTCTCTCTTCGTCTTCCTCGGCCGTAGCGCGAAGAAGAACCGCCGGCATTCAGCATTCTGACCCTAGACACAGAGTCCTCCGGTCTTCTGATAAACCCCCATGTGGAAGGCCAAGAC      120
2       TTTCTCTCTTCGTCTTCCTCGGCCGTAGCGCGAAGAAGAACCGCCGGCATTCAGCATTCTGACCCTAGACACAGAGTCCTCCGGTCTTCTGATAAACCCCCATGTGGAAGGCCAAGAC      120
        ************************************************************************************************************************

1       ATGCTTCCGTCAGATTTACTTGACCGTACTAATACGGCGGTACTCGAGAGTCGCTCCGCCGCCGTCTTCGGTGATCCGCGTGACAAACAACGTAGCACACCTGGGACCACCGAAGCAAGG      240
2       ATGCTTCCGTCAGATTTACTTGACCGTACTAATACGGCGGTACTCGAGAGTCGCTCCGCCGCCGTCTTCGGTGATCCGCGTGACAAACAACGTAGCACACCTGGGACCACCGAAGCAAGG      240
        ************************************************************************************************************************

1       ACCACTGCCACGTCAGCTGATATCCCTGCCGCCATTTCCCGGTCATCCATTACCTGGCAAAAACGCCGGAGCTGACGGCGACGATGGAGATAGCGGCGGCCACGTCACAGCTATAAGCTG      360
2       ACCACTGCCACGTCAGCTGATATCCCTGCCGCCATTTCCCGGTCATCCATTACCTGGCAAAAACGCCGGAGCTGACGGCGACGATGGAGATAGCGGCGGCCACGTCACAGCTATAAGCTG      360
        ************************************************************************************************************************

1       GGTCAAGTACTATTTTGAAGAAATCTATGATAAGGCTATTCAAACTCATTTCACAAAGGGCCTT-------------GTTCAGATGGAGTTTCGAGGTCGTAGGGATGCTTCAAGAGAGA      467
2       GGTCAAGTACTATTTTGAAGAAATCTATGATAAGGCTATTCAAACTCATTTCACAAAGGGCCTTGTGAGTGTCTCTGGTTCAGATGGAGTTTCGAGGTCGTAGGGATGCTTCAAGAGAGA      480
        ****************************************************************             *******************************************

1       AAGAAGATGGAGCTATTCCTATGAGAAAGATTAAGCATAACGAGGTGATGCAAATAGGAGACAAAATCTGGTTGCCGGTTTCAATCGCTGAGATGAGGATTTCTAAGAGATATGACACCA      587
2       AAGAAGATGGAGCTATTCCTATGAGAAAGATTAAGCATAACGAGGTGATGCAAATAGGAGACAAAATCTGGTTGCCGGTTTCAATCGCTGAGATGAGGATTTCTAAGAGATATGACACCA      600
        ************************************************************************************************************************

1       TACCAAGTGGAACCTTGTATCCAAACGCAGACGAAATCGCATATCTTCAAAGGCTTGTCAGGTTCAAGGACTCTGCTATTATAGTTCTTAATAAGCCACCTAAGCTTCCAGTCAAGGGAA      707
2       TACCAAGTGGAACCTTGTATCCAAACGCAGACGAAATCGCATATCTTCAAAGGCTTGTCAGGTTCAAGGACTCTGCTATTATAGTTCTTAATAAGCCACCTAAGCTTCCAGTCAAGGGAA      720
        ************************************************************************************************************************

1       ATGTGCCTATACATAATAGCATGGATGCACTTGCAGCTGCAGCTTTGTCTTTTGGTAACGATGAAGGTCCTAGATTGGTACATCGTCTTGATAGGGAAACTAGTGGCCTCTTAGTAATGG      827
2       ATGTGCCTATACATAATAGCATGGATGCACTTGCAGCTGCAGCTTTGTCTTTTGGTAACGATGAAGGTCCTAGATTGGTACATCGTCTTGATAGGGAAACTAGTGGCCTCTTAGTAATGG      840
        ************************************************************************************************************************

1       GTCGAACCAAAGAAAGTATAGATTATCTTCACTCAGTGTTCAGTGACTACAAGGGGAGAAACTCAAGCTGTAAGGCTTGGAACAAAGCGTGTGAGGCGATGTATCAGCAATATTGGGCAT      947
2       GTCGAACCAAAGAAAGTATAGATTATCTTCACTCAGTGTTCAGTGACTACAAGGGGAGAAACTCAAGCTGTAAGGCTTGGAACAAAGCGTGTGAGGCGATGTATCAGCAATATTGGGCAT      960
        ************************************************************************************************************************

1       TGGTGATTGGTTCTCCAAAGGAAAAAGAAGGACTAATTTCAGCTCCTCTTTCAAAGGTGCTTTTGGACGATGGTAAAACAGACAGGGTGGTTTTGGCTCAAGGTTCGGGCTTTGAAGCTT      1007
2       TGGTGATTGGTTCTCCAAAGGAAAAAGAAGGACTAATTTCAGCTCCTCTTTCAAAGGTGCTTTTGGACGATGGTAAAACAGACAGGGTGGTTTTGGCTCAAGGTTCGGGCTTTGAAGCTT      1020
        ************************************************************************************************************************
```

```
1     CGCAAGATGCAATAACAGAGTATAAAGTGTTAGGACCTAAGATCAACGGGTGTTCGTGGGTAGAACTTCGTCCTATTACTAGCAGAAAACATCAGCTACGTGTACACTGCGCTGAAGCAC     1187
2     CGCAAGATGCAATAACAGAGTATAAAGTGTTAGGACCTAAGATCAACGGGTGTTCGTGGGTAGAACTTCGTCCTATTACTAGCAGAAAACATCAGCTACGTGTACACTGCGCTGAAGCAC     1200
      ************************************************************************************************************************

1     TTGGTACTCCAATAGTAGGGGATTACAAGTACGGTTGGTTTGTTCACAAGAGATGGAAACAGATGCCTCAGGTTGATATCGAACCAACTACTGGGAAACCATATAAACTGCGCAGACCAG     1307
2     TTGGTACTCCAATAGTAGGGGATTACAAGTACGGTTGGTTTGTTCACAAGAGATGGAAACAGATGCCTCAGGTTGATATCGAACCAACTACTGGGAAACCATATAAACTGCGCAGACCAG     1320
      ************************************************************************************************************************

1     AAGGTCTTGATGTCCAAAAGGGAAGCGTTTTGTCAAAAGTACCTTTGTTACATCTCCATTGCCGGGAAATGGTACTTCCAAACATTGCCAAGTTCCTACATGTCATGAACCAACAGGAAA     1427
2     AAGGTCTTGATGTCCAAAAGGGAAGCGTTTTGTCAAAAGTACCTTTGTTACATCTCCATTGCCGGGAAATGGTACTTCCAAACATTGCCAAGTTCCTACATGTCATGAACCAACAGGAAA     1440
      ************************************************************************************************************************

1     CAGAGCCGCTTCACACAGGAATCATTGATAAACCGGATCTCTTGCGGTTTGTAGCTTCAATGCCCAGCCATATGAAGATCAGTTGGAACTTAATGTCTTCATATTTGGTGTAGCTCTTTT     1547
2     CAGAGCCGCTTCACACAGGAATCATTGATAAACCGGATCTCTTGCGGTTTGTAGCTTCAATGCCCAGCCATATGAAGATCAGTTGGAACTTAATGTCTTCATATTTGGTGTAG-------     1553
      *****************************************************************************************************************

1     ACTACCTTTTTATTATTAGATAACAGTTCCTGAACTTCTTGGTTCTGTGGCTTCATATTGGGAATATTTGTATTGTACAGTTGTCTCACAGA                                 1639
2     --------------------------------------------------------------------------------------------                                 1553
```

Figure S2. Protein and nucleotide sequence comparison of the validated gene *AT1G78910* (*PUS3*). (A) Alignment of the predicted protein sequences of the two PUS3 isoforms, highlighting amino acid differences that result from AS. (B) Alignment of the corresponding nucleotide sequences, illustrating the splice-site variation that underlies the observed protein differences.

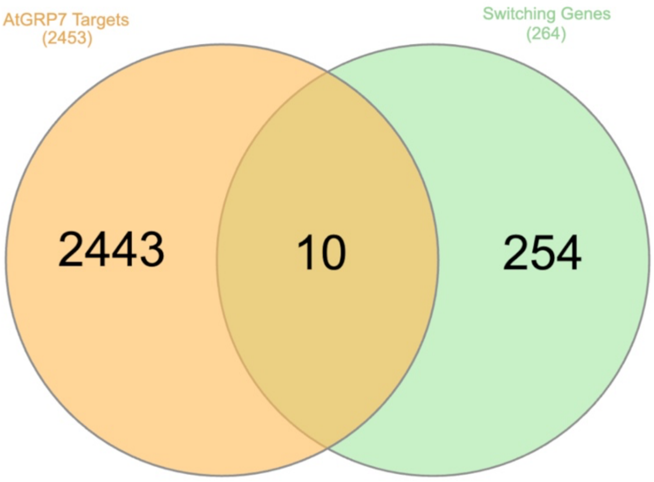

Figure S3. Overlap of isoform-switching genes with AtGRP7 RIP-seq targets. Venn diagram comparing isoform-switching genes from this study (264 genes) with AtGRP7 targets identified by RNA immunoprecipitation (RIP-seq; 2,453 transcripts; Meyer et al., 2017). Only ten genes overlapped, indicating that the enrichment observed for SR45-dependent targets is not a general feature of RNA-binding proteins but reflects a specific regulatory role.

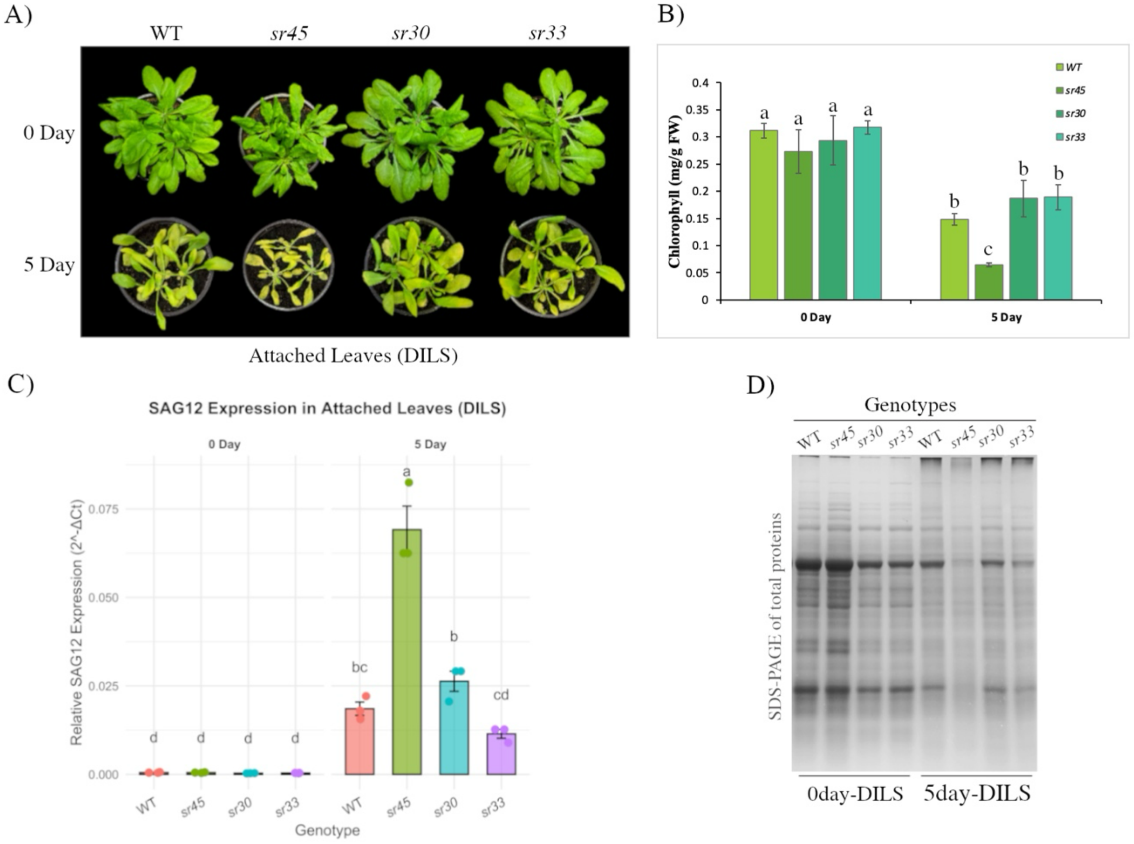

Figure S4. Comparative analysis of SR mutants in the DILS assay. (A) Phenotypes of attached leaves from WT, *sr45*, *sr30*, and *sr33* plants at day 0 and after 5 days of DILS. (B) Chlorophyll content (mg g^{-1} FW) in attached leaves of the indicated genotypes at 0 and 5 days. Different letters denote significant differences (ANOVA, Tukey's HSD, $p < 0.05$). (C) Expression of the senescence marker gene *SAG12* (qRT–PCR, relative to *ACT2*) in attached leaves at day 0 and day 5. Data are means ± SE; letters indicate statistical groups ($p < 0.05$). (D) Total protein profiles of attached leaves from the indicated genotypes at 0 and 5 days of DILS, analyzed by SDS–PAGE.
